# Altered conformational dynamics contribute to species-specific effects of cytochrome *c* mutations on caspase activation

**DOI:** 10.1007/s00775-024-02044-2

**Published:** 2024-03-12

**Authors:** Thomas C. Chin, Sigurd M. Wilbanks, Elizabeth C. Ledgerwood

**Affiliations:** Biochemistry Department, School of Biomedical Sciences, University of Otago, Dunedin, New Zealand

**Keywords:** Cytochrome *c*, Apoptosis, Molecular dynamics, Peroxidase, Apaf-1

## Abstract

Variants in the gene encoding human cytochrome *c* (*CYCS*) cause mild autosomal dominant thrombocytopenia. Despite high sequence conservation between mouse and human cytochrome *c*, this phenotype is not recapitulated in mice for the sole mutant (G41S) that has been investigated. The effect of the G41S mutation on the in vitro activities of cytochrome *c* is also not conserved between human and mouse. Peroxidase activity is increased in both mouse and human G41S variants, whereas apoptosome activation is increased for human G41S cytochrome *c* but decreased for mouse G41S cytochrome *c*. These apoptotic activities of cytochrome *c* are regulated at least in part by conformational dynamics of the main chain. Here we use computational and in vitro approaches to understand why the impact of the G41S mutation differs between mouse and human cytochromes *c*. The G41S mutation increases the inherent entropy and main chain mobility of human but not mouse cytochrome *c*. Exclusively in human G41S cytochrome *c* this is accompanied by a decrease in occupancy of H-bonds between protein and heme during simulations. These data demonstrate that binding of cytochrome *c* to Apaf-1 to trigger apoptosome formation, but not the peroxidase activity of cytochrome *c*, is enhanced by increased mobility of the native protein conformation.

**Supplementary Information:**

The online version contains supplementary material available at 10.1007/s00775-024-02044-2.

## Introduction

Cytochrome *c* is an essential electron carrier in mitochondrial respiration and also an important mediator of the intrinsic apoptosis pathway [1, 2]. In the intrinsic apoptosis pathway, cytochrome *c* is released from mitochondria into the cytosol in response to stress stimuli. In the cytosol, cytochrome *c* interacts with apoptotic protease activating factor 1 (Apaf-1) triggering formation of the apoptosome and subsequently caspase activation [3, 4]. During apoptosis cytochrome *c* is also proposed to be converted to a peroxidase-active conformer, which contributes to its own release into the cytosol by catalyzing the oxidation of cardiolipin [1, 5].

Five naturally occurring variants in human cytochrome *c* have been reported (c.79C > T, p.His27Tyr; c.132G > A, p.Gly42Ser; c.145 T > C, pTyr49His; c.155C > T, pAla52Val, and c.301_303del:p.Lys101del^1^), all associated with mild autosomal dominant thrombocytopenia (THC4 MIM 612004) [6–10]. The G41S, Y48H and A51V variants have been shown to enhance caspase activation and peroxidase activities of cytochrome *c* in vitro [6, 11–15]. Although the overall structure of cytochrome *c* is unchanged in these three variants, the mutations cause a decrease in global stability and a lowered alkaline isomerization p*K* [11, 13, 14, 16]. The underlying molecular mechanisms for these changes are not fully understood, although increased protein dynamics has a role [15–17].

**Fig. 1:**
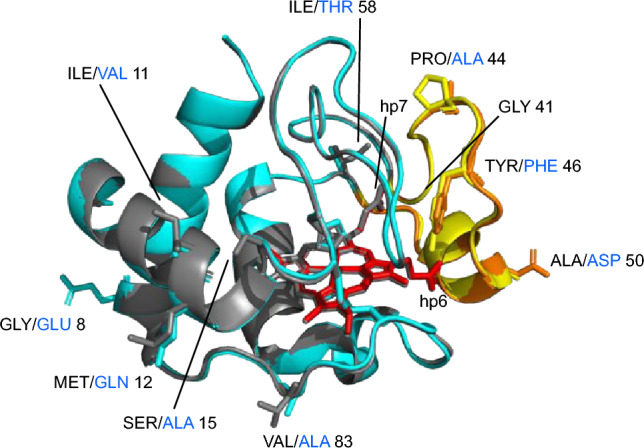
Structural alignment of mouse and human cytochromes *c*. Mouse (cyan) and human (grey) cytochrome *c* structures are superimposed with the 40–57 Ω-loops coloured in orange (mouse) and yellow (human). The heme is coloured red (mouse) and grey (human). Residue differences between structures shown as sticks labelled with three-letter residue codes (black for human, blue for mouse) and residue position number. Heme propionates (hp) 6 and 7 and the position of Gly41 are also shown. Drawn from PDB entries 3ZCF (human) and 5C0Z (rat, which has an identical sequence to mouse)

Cytochrome *c* is highly conserved [18], however, the effects of cytochrome *c* mutations are species specific, both in vivo and in vitro. Despite 91% sequence identity between human and mouse cytochromes *c* (Fig. 1), homozygous G41S knockin mice do not recapitulate the thrombocytopenia phenotype of humans [19]. Further investigation showed that recombinant mouse G41S cytochrome *c* has increased peroxidase activity as seen with human G41S cytochrome *c*; whereas, in contrast to human G41S cytochrome *c,* there was a decreased ability of mouse G41S cytochrome *c* to activate caspases [12, 19]. Three of the nine sequence differences between human (hu) and mouse (ms) cytochromes *c* are in the 40–57 Ω loop (Fig. 1) which has been implicated in controlling the peroxidase activity and alkaline isomerisation p*K* of cytochrome *c* [20]. This loop contains three of the human variants associated with thrombocytopenia (G41S, Y48H and A51V), with another human variant making a H-bond to this loop (H26Y). CryoEM studies of the ground and active apoptosome place the 40–57 Ω loop in the Apaf-1—cytochrome *c* binding interface but these structures do not have sufficient resolution to refine mainchain conformation or sidechain orientations of cytochrome *c* [21–23]. Previous modelling identified five potential polar bonds between the 40–57 Ω loop and the Apaf-1 WD2 domain [15]. The residues involved in these bonds are conserved between mouse and human cytochrome *c* and Apaf-1, so do not provide a possible explanation of the difference in the functional impact of the G41S mutation in human vs mouse cytochrome *c*.

We have previously carried out in vitro and in silico analyses of pathological and non-natural human cytochrome *c* variants (wildtype (WT), G41S, Y48H, A51V, G41T and G41A) [15]. In molecular dynamics (MD) simulations of the native protein the majority of mainchain atoms showed RMSFs of less than 0.6 Å, with omega loops rising to 1.5 Å, while those of some mutant proteins showed ‘increased mobility’ with RMSFs of greater than 1.5 Å. Variation in caspase activation but not peroxidase activity correlated with conformational mobility, particularly in the 19–36 and 40–57 Ω-loops. Here we hypothesize that the distinct functional effect of the G41S mutation on caspase activation in mouse compared to human cytochrome *c* is due to a different impact on the conformational dynamics of the protein main chain. To test this, we perform in silico and in vitro analyses of mouse WT and G41S cytochromes *c* to compare with human WT and G41S cytochromes *c*. These data show that while the G41S mutation has an impact on the mobility of human cytochrome *c*, this is not the case for mouse cytochrome *c*, and the results provide additional evidence that mutation-driven increases in caspase activation, but not peroxidase activity, occur due to increased main chain dynamics.

## Materials and methods

### Molecular dynamics

Molecular dynamics (MD) simulations of mouse WT and G41S cytochromes *c* were performed essentially as described previously for human cytochrome *c* [15]. The structure of recombinant mouse oxidized cytochrome *c* is available in the Protein Database (PDB 5C0Z), identified as rat cytochrome *c* which has an identical sequence [24]. The 5C0Z chain A structure was modified by addition of the serine 41 sidechain in PyMol (PyMOL Molecular Graphics System, Schrödinger, LLC) to obtain the starting model for mouse G41S cytochrome *c*. Each of the two starting models were used in four simulations, two each using parameters for the oxidized (denoted FeIII_1 and FeIII_2) and reduced heme (denoted FeII_1 and FeII_2) forms [25]. In brief, structures were solvated with TIP3P water while centred in a box approximately 66 Å × 61 Å × 66 Å and total charge neutralized with seven or eight chloride atoms. The protonation state for each model was set at pH 7.0 [26] using the PDB2PQR webserver [27]. Hydrogen atoms were removed from the models before proceeding. The VMD [28] packages autoionize and solvate, and cytochrome *c* specific topology files [25], were used to prepare the models for the simulations. The simulations were performed at NeSI (NZ eScience Infrastructure). All simulations were done with NAMD (v 2.12) using CHARMM36 potentials [29–31]. Under these conditions the heme remains hexacoordinate. Simulations used a time step of 2 fs with data saved every 10 ps. Simulations ran for 200 ns, not including an initial equilibration of 16.5 ns, with each simulation run in duplicate at each oxidation state. MD data for human WT and G41S cytochromes *c* were previously described [15]. Hydrogen bond occupancy was determined using the Hydrogen Bond package in VMD [28], with settings of 3.2 Å for donor acceptor distance, 30º for angle cut-off [32], and polar atoms only. H-bond occupancies were calculated for residue-residue, residue-heme and residue-water bonds. Solvent accessible surface area (SASA) was determined in VMD with SASA.tcl [https://www.ks.uiuc.edu/Research/vmd/mailing_list/vmd-l/att-18670/sasa.tcl].

### Expression, purification and characterization of cytochrome c variants.

Mouse and human WT and G41S cytochrome *c* variants were expressed and purified as previously described [33], and the presence of each mutation was confirmed by intact mass analysis on a 4800 MALDI-TOF analyzer (Centre for Protein Research, University of Otago). The concentration of ferricytochrome *c* was measured using an extinction coefficient of 106.1 mM^−1^ cm^−1^ at 410 nm.

### Circular dichroism (CD) spectroscopy

Purified ferricytochrome *c* was dialysed into 50 mM citric acid buffer pH 3.8 (thermal denaturation studies) or 50 mM sodium phosphate pH 7.5 (spectra) and diluted to 20 μM. CD spectroscopy was performed using a JASCO J-1500 spectrophotometer with a 1 mm pathlength quartz cuvette. Spectra were recorded at 25 °C from 260 to 200 nm with a 1.0 nm step size and a slit width of 1.5 nm. Digital integration time was 4 s/point and ellipticities reported as molar ellipticity in deg cm^2^∙dmol^−1^. Single replicates of thermal denaturation profiles were acquired by monitoring the ellipticity at 222 nm as a function of temperature between 4 and 96 °C in 2 °C increments at a rate of 1 °C min^−1^. Plots of spectra and thermal denaturation profiles are in Figure S1. Cytochrome *c* undergoes a two-state transition upon unfolding [34], and the midpoint of thermal denaturation (*T*_*m*_), ΔH and ΔS were determined using a N ⇌ D two-state model in CDPal [35], fixing ΔCp at 6.69 kJ mol^−1^ K^−1^ as reported for human cytochrome *c* [36]. Note that the same results were obtained when ΔCp was fixed at 0 kJ mol^−1^ K^−1^. The uncertainties of the fits were determined with Jackknife in CDPal.

## Results and discussion

### Mutation of glycine 41 to serine alters the conformational mobility of human but not mouse cytochrome c

In this study, we performed four independent 200 ns simulations for ms WT and G41S cytochromes *c*, using PDB 5C0Z, identified as rat oxidized rat cytochrome *c* but the same amino acid sequence as mouse cytochrome *c*. Apoptosome activation assays use reduced cytochrome *c* whereas peroxidase activity requires oxidized cytochrome *c*. Therefore, for each variant, two simulations incorporated an oxidized heme (FeIII_1 and FeIII_2) and two a reduced heme (FeII_1 and FeII_2). Under the parameters used in the simulations the ligation between Met80 and the heme Fe stays intact. We compared these simulations with those previously reported for human WT and G41S cytochromes *c* [15].

**Fig. 2:**
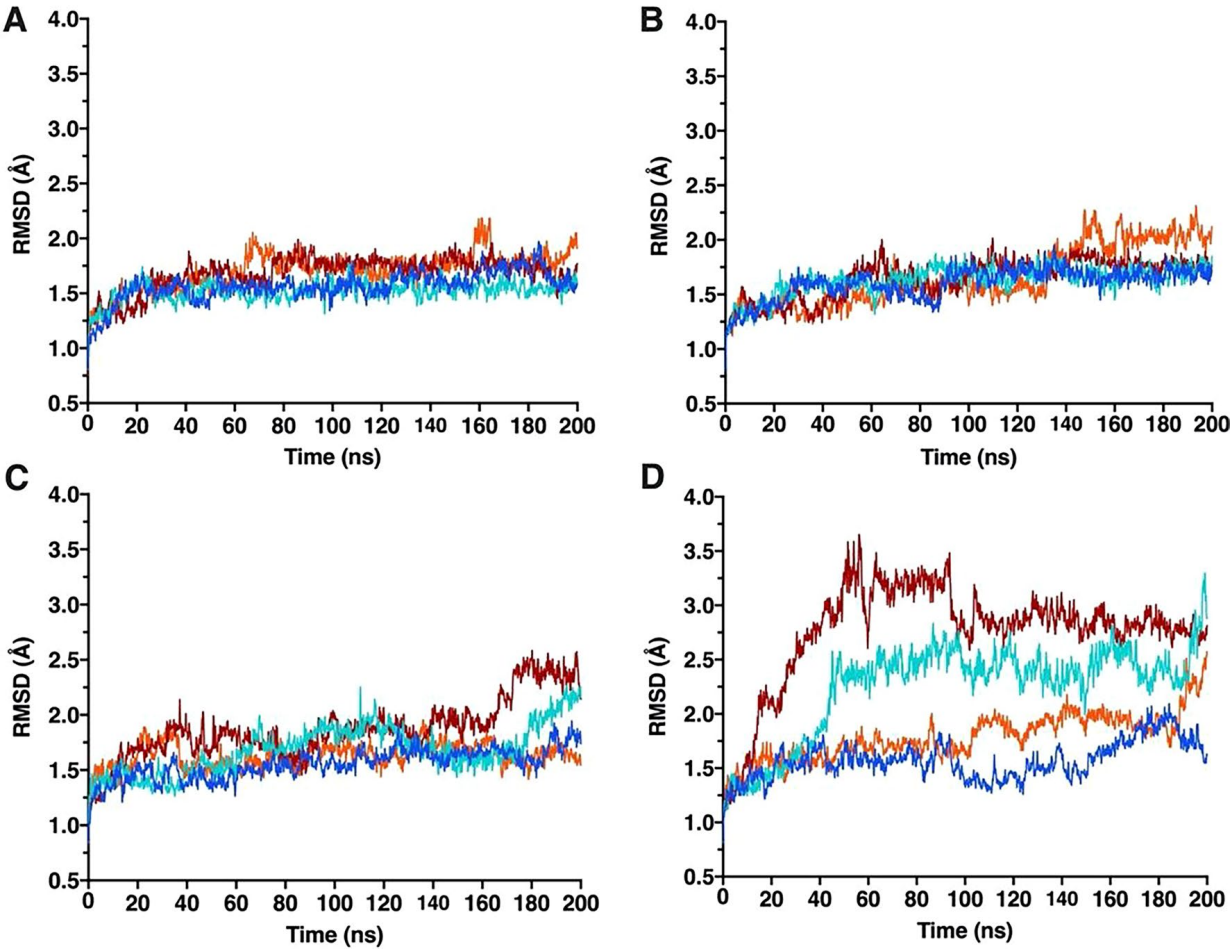
Mainchain movement of mouse and human cytochrome *c* variants. Root mean squared deviation (RMSD) (Å) between the 5C0Z (mouse) or 3NWV (human) starting structure and the states captured during the MD simulations for each cytochrome c variant. **A** Mouse WT cytochrome *c* runs. **B** Mouse G41S cytochrome *c* runs. **C** Human WT cytochrome *c* runs. **D** Human G41S cytochrome *c* runs. FeII_1 runs are shown in blue, FeII_2 in cyan, FeIII_1 in dark red, FeIII_2 in orange

To determine the effect of the G41S mutation on the main chain mobility of mouse and human cytochromes *c*, we analyzed root mean squared deviation (RMSD) over the 200 ns simulations. Both WT cytochromes *c* have a stable RMSD over time (Fig. 2 A and C), consistent with this small globular protein maintaining the starting main chain fold throughout the simulation. The G41S mutation increases main chain RSMD in human but not mouse cytochrome *c* (Fig. 2 B and D). In two simulations (FeII_2 and FeIII_1) human G41S cytochrome *c* populates states that are not occupied by msWT, msG41S or huWT cytochromes *c*. As previously reported, the increased RMSD of huG41S cytochrome *c* is driven primarily by movement of the 40–57 Ω loop [15].

The solvent accessible surface area (SASA) of both the total protein and the heme was evaluated to determine whether the increased RMSD in the huG41S simulations corresponds with a more open conformation and/or altered heme accessibility (Table 1). Mouse WT and G41S, human WT and the lower RMSD human G41S simulations (FeII_1 and FeIII_2) have similar protein SASA. For the two higher RMSD human G41S simulations (FeII_2 and FeIII_1) SASA is increased, consistent with the 40–57 Ω loop moving away from the heme as previously reported [15]. However, there is little change in solvent accessibility of the heme in any simulation. Taken together these results show that mutation of glycine 41 to serine increases the mobility of the main chain in human but not mouse cytochrome *c*.

**Table 1:** Average SASA for mouse and human cytochrome *c* variants

	msWT	msG41S	huWT	huG41S			
				FeII_1	FeII_2	FeIII_1	FeIII_2
Protein	6710 ± 140	6780 ± 140	6860 ± 160	6860 ± 120	7220 ± 170	7290 ± 160	7000 ± 140
Heme	829 ± 9	829 ± 9	835 ± 20	857 ± 9	851 ± 18	835 ± 10	830 ± 19

Average SASA (Å^2^ ± SD) was calculated between 40 and 160 ns across all four cytochrome *c* MD simulations (FeII_1, FeII_2, FeIII_1 and FeIII_2) for msWT, msG41S and huWT, and for individual runs for huG41S

### Mutation of glycine 41 to serine alters thermodynamic properties underlying the stability of human but not mouse cytochrome c

**Fig. 3:**
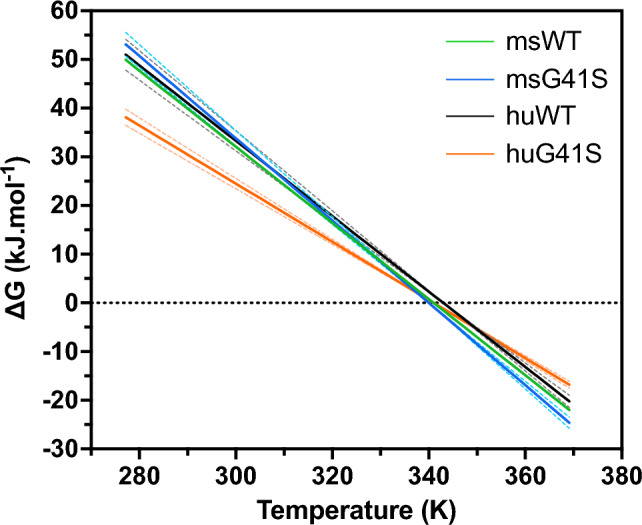
Gibbs–Helmholtz plot of mouse and human WT and G41S cytochromes *c*. Gibbs–Helmholtz plot calculated with the unfolding transition data for msWT (green), msG41S (blue), huWT (black) and huG41S (orange) from Table 2. The dashed lines in matching colour represent the errors reported in Table 2. The Gibbs–Helmholtz plot is defined by the equation: ∆G = ∆H(1-*T*/*T*_*m*_). Dashed line at ∆G = 0, where unfolding/refolding is neither favourable nor unfavourable

We used CD spectroscopy to investigate whether the increased main chain mobility reported by the MD simulations for huG41S cytochrome *c* decreased the protein’s thermal stability. The thermodynamic parameters (Table 2) for human WT and G41S cytochrome *c* are consistent with those reported previously [13, 36, 37]. Under conditions in which unfolding is reversible (pH 3.8 [36]), the G41S mutation had little impact on overall thermodynamic stability (assessed by melting temperatures (*T*_*m*_)) in either mouse or human cytochrome *c*. However, the underlying thermodynamic parameters of unfolding differ. These were extracted from the thermal denaturation profiles and displayed on a Gibbs–Helmholtz plot (Fig. 3). The msWT, msG41S and huWT cytochromes *c* are very similar whereas huG41S cytochrome *c* shows a flatter curve, reflecting the lower ΔH and ΔS values and a smaller ΔG of unfolding at body temperature. The similar *T*_*m*_ of huG41S results from compensatory decreases in both ∆S (the slope) and ∆H (vertical intercept). Overall, these results suggest both a greater entropy of the native state for huG41S, and lower stability at physiologically relevant temperature, consistent with the higher main chain dynamics reported in the simulations.

**Table 2:** Thermodynamic parameters for cytochrome *c* unfolding

Variant	*T*_*m*_ (°C)	∆H (kJ.mol^−1^)	∆S (kJ.K^−1^.mol^−1^)
Mouse WT	67.8 ± 0.3	267 ± 13	3.94 ± 0.19
Mouse G41S	66.9 ± 0.2	287 ± 13	4.29 ± 0.20
Human WT	69.3 ± 0.3	266 ± 17	3.83 ± 0.24
Human G41S	67.6 ± 0.2	204 ± 9	3.01 ± 0.13

The uncertainties for T_m_, ∆H and ∆S are for the the fits in CDPal, estimated using Jackknife

### Mutation of glycine 41 to serine promotes changes in the H-bond network of cytochrome c corresponding with differences in conformational dynamics between species

It is intriguing that despite a high degree of sequence conservation between mouse and human cytochrome *c*, mutation of Gly41 to Ser alters the dynamics of only human cytochrome *c*. We hypothesized that this is due to differences in the hydrogen bond (H-bond) networks linking the Ω loops to the propionate groups of the heme in human and mouse cytochrome *c*. In the crystal structure of huG41S cytochrome *c*, the hydroxyl of Ser41 interacts with the sidechain nitrogen of Asn52, altering the H-bond network linking the 40–57 Ω loop to the heme [15, 33], but no structural data is available for msG41S cytochrome *c.* Therefore, to address this hypothesis, we extracted the occupancies of all residue∙∙∙residue, residue∙∙∙heme and residue∙∙∙water H-bonds from each MD simulation between 40 and 160 ns, over which time all simulations had a relatively stable RMSD. We were unable to directly compare the H-bonds in the simulations to the crystal structure, as some water molecules observed in the Ω loop—heme networks in crystal structure (determined at 100 K) were not observed in the simulations (carried out at 298 K). Instead, we compared substantially similar conformations within the simulations, identifying H-bonds with a difference in occupancy in the huG41S simulations with low RMSD (FeII_1 and FeIII_2) compared to huWT, msWT and msG41S. Our rationale was that this could identify intrinsic differences in huG41S that would explain the increased huG41S mobility in the other two simulations (FeII_2 and FeIII_1).

**Fig. 4:**
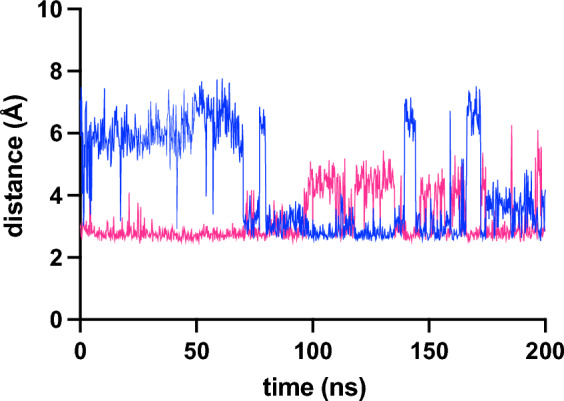
Selected residue distances for HuG41S FeII_1 simulation. Distance (Å) over huG41S FeIII_1 simulation for Asn52δO to Thr78OG1 (blue) and Thr78γO to the closest oxygen of hp6 (pink)

Occupancies for the majority of the residue∙∙∙residue, residue∙∙∙heme and residue∙∙∙water H-bonds were very similar across all simulations (Supplementary Data 1–3) as expected from the low RMSD. However, a difference in occupancy of H-bonds linking the 40–57 and 70 s Ω loops with the heme was identified (Table 3). In both WT proteins and msG41S the 40–57 and 70 s Ω loops are anchored to the heme propionates via medium–high occupancy H-bonds to the side chains of Asn52 and Thr78, respectively. Thr78 forms a H-bond with heme propionate-6 whereas Asn52 forms a H-bond with both heme propionate-6 or -7, consistent with NMR and crystallography data [33, 38]. Mutation of Gly41 to Ser in human cytochrome *c* is associated with a decreased in occupancy of these H-bonds, and a new H-bond links the sidechains of Asn52 and Thr78. By examining the trajectory of these H-bonds in a huG41S simulation it is apparent that the formation of the Asn52∙∙∙Thr78 H-bond is mutually exclusive with H-bonds linking Thr78 to the heme propionate (Fig. 4). Overall, these changes in H-bond occupancy suggest that in the context of human but not mouse cytochrome *c*, mutation of residue 41 to serine destabilizes the connection between the 40–57 and 70 s Ω loops and the heme propionates. As a result, either the new Asn52∙∙∙Thr78 H-bond forms (as seen in the huG41S FeII-1 and FeIII_2 simulations), or the 40–57 Ω loop unfolds (as seen in the huG41S FeII_2 and FeIII_1 simulations).

**Table 3:** H-bond occupancy

	msWT		msG41S		huWT		huG41S	
	FeII (%)	FeIII (%)	FeII (%)	FeIII (%)	FeII (%)	FeIII (%)	FeII_1 (%)	FeIII_2 (%)
Asn52δN∙∙∙hp6^1^	48	35	36	37	29	31	3	11
Asn52δN∙∙∙hp7^2^	10	29	0	13	14	40	12	2
Thr78γO∙∙∙hp6^3^	87	75	65	38	82	60	39	18
Thr78γO∙∙∙Asn52δO	0	1	4	19	3	4	32	33

^1^sum of occupancy of H-bonds to both oxygen atoms of heme propionate 6 (hp6)

^2^sum of occupancy of H-bonds to both oxygen atoms of heme propionate 7 (hp7)

^3^sum of occupancy of H-bonds to both oxygen atoms of hp6

H-bond occupancy between 40 and 160 ns for msWT, msG41S and huWT was calculated across the combined FeII and FeIII simulations for each protein. Data for two individual simulations are shown for huG41S. To account for flipping of the propionate we report the sum of occupancies of the H-bonds to the two oxygens

The question remains as to why the presence of serine at residue 41 in human cytochrome *c* destabilizes the Asn52/Thr78∙∙∙heme H-bonds sufficiently to allow loop unfolding while G41S in mouse cytochrome *c* does not. We considered whether the Ser41∙∙∙Asn52 H-bond present in the huG41S crystal structure contributed to the mouse-human difference. However, this H-bond was not well populated (0–13% occupancy in mouse and human G41S cytochromes *c*). Presumably the mouse-human sequence differences in the 40–57 Ω loop stabilize the Asn52∙∙∙heme H-bond when residue 41 is serine in mouse cytochrome *c*, and thus prevent unfolding. We have previously reported that substitution of Ala44 in mouse cytochrome *c* with the Pro seen in human cytochrome *c* does not humanize the impact of the G41S substitution on caspase activation [19]. A human cytochrome *c* variant with Tyr46 substituted with the mouse Phe has also been studied [11]. The Tyr46 phenolic oxygen in involved in a network connecting the surface Ω loops, with H-bonds to a water molecule and the main chain of Thr28. These H-bonds are lost with Phe46, but it is unclear how loss of these bonds would prevent the destabilizing effect of the G41S mutation on the 40–57 Ω loop. Furthermore, occupancy of the Tyr46∙∙∙water and Tyr46∙∙∙Thr28 bonds does not change between WT, the stable and the unstable huG41S simulations (Supplementary Data 1 and 3), implying that the Tyr46 centred H-bond network does not contribute to the stability of the 40–57 Ω loop.

## Conclusion

The simulation of the dynamics of human and mouse WT and G41S cytochromes *c* extends our understanding of the role of protein dynamics in controlling the pro-apoptotic activities of cytochrome *c*. Introduction of serine at residue 41 has previously been shown to increase the mobility of human cytochrome *c* as assessed by both molecular dynamics and HDX NMR [15, 17]. In contrast we show here that the main chain mobility of mouse cytochrome *c* does not change when serine is introduced at residue 41. Although human G41S and WT cytochromes *c* have similar global stability, consistent with previous reports [11, 37], unfolding of human G41S has a lower ΔS, suggesting a higher entropy in the native state. This appears to be driven by the changes in the strength of the H-bonds linking Asn52 and Thr78 with the heme propionates. Asn52 has previously been identified as an important residue for both the folding and the stability of cytochrome *c* [39].

Replacement of Gly41 by Ser increases apoptosome activation by human but not mouse cytochrome *c*. The increase in mobility of the hexacoordinate native state of human compared to mouse G41S cytochrome *c* supports the view that movement of the 40–57 Ω loop facilitates binding to Apaf-1 to trigger apoptosome formation. In contrast, since peroxidase activity is higher than WT for both human and mouse G41S cytochrome *c*, it is unlikely that increased mobility of the main chain in the hexacoordinate state is required for conversion to the pentacoordinate peroxidase-active state.

### Supplementary Information

Below is the link to the electronic supplementary material.

Supplementary file1 (XLSX 113 KB)

## Data Availability

The datasets generated during and/or analysed during the current study are available from the corresponding author on reasonable request.

## Footnotes

1 Note that the amino acid numbering in the formal description of each genetic variant refers to the protein with the initiating methionine as residue 1. Subsequently we refer to the proteins using numbering for the mature protein, i.e. G41S, Y48H, A51V, H26Y.
